# Effects of Dietary CpG Oligodeoxynucleotides (CpG ODNs) Supplementation Levels on Growth Performance, Immunity, Digestive Capacity, Intestinal Microbiota, and Transcriptomic Response in *Litopenaeus vannamei*

**DOI:** 10.3390/ani16142207

**Published:** 2026-07-16

**Authors:** Hongming Wang, Cuihong Hou, Yudong Zheng, Hang Yuan, Beiping Tan, Lili Shi, Shuang Zhang

**Affiliations:** 1 College of Fisheries, Guangdong Ocean University, Zhanjiang 524088, China; wanghongming97@163.com (H.W.); houcuihong17@163.com (C.H.); yd_zheng96@126.com (Y.Z.); yh17817895172@163.com (H.Y.); 13590054296@163.com (L.S.); 2 Key Laboratory of Aquatic, Livestock and Poultry Feed Science and Technology in South China, Ministry of Agriculture, Zhanjiang 524088, China; 3 Aquatic Animals Precision Nutrition and High Efficiency Feed Engineering Research Center of Guangdong Province, Zhanjiang 524088, China

**Keywords:** CpG ODNs, immunity, intestinal microbiota, transcriptome, *Litopenaeus vannamei*

## Abstract

This study evaluated the effects of dietary CpG oligodeoxynucleotide (CpG ODNs) on growth performance, immunity, digestive function, intestinal microbiota, and transcriptomic responses in *Litopenaeus vannamei*. Shrimp with an initial body weight of 0.30 ± 0.02 g were fed diets supplemented with 0, 0.1, 0.4, 1.6, 6.4, or 25.6 mg kg^−1^ CpG ODNs for 8 weeks. Dietary CpG ODNs did not significantly improve most growth parameters; however, shrimp fed the diet supplemented with 1.6 mg kg^−1^ CpG ODNs showed a lower feed conversion ratio. After white spot syndrome virus challenge, shrimp fed the diet supplemented with 25.6 mg kg^−1^ CpG ODNs showed higher survival than those in the control group, whereas survival after *Vibrio* parahaemolyticus challenge did not differ significantly among treatments. Dietary supplementation with CpG ODNs at levels above 0.4 mg kg^−1^ increased the activities of several serum immune-related and antioxidant enzymes, reduced malondialdehyde content, and enhanced intestinal trypsin and amylase activities. In addition, this treatment increased microbial richness indices, while reducing the relative abundances of *Vibrio* and *Photobacterium*. Transcriptomic analysis showed that several pathways, including the AMPK signaling pathway and JAK–STAT signaling pathway, were significantly enriched in the CpG ODNs (above 0.4 mg kg^−1^) groups compared with the control group. In conclusion, dietary supplementation with CpG ODNs did not significantly improve the growth performance of *L. vannamei*. However, dietary supplementation with CpG ODNs at levels above 0.4 mg kg^−1^ may help enhance immune and digestive functions and modulate the intestinal microbiota composition of *L. vannamei*.

## Simple Summary

*Litopenaeus vannamei* are frequently affected by infectious diseases and intestinal imbalance during intensive farming. This study tested whether dietary CpG oligodeoxynucleotide (CpG ODNs) could improve shrimp health. The results showed that CpG ODNs supplementation did not significantly improve the growth performance of *L. vannamei*. However, dietary supplementation with appropriate levels of CpG ODNs enhanced immune and digestive enzyme activities, improved resistance to white spot syndrome virus challenge, and modulated intestinal microbiota composition. These findings suggest that CpG ODNs may be a potential health-promoting functional feed additive, although its optimal dose and mechanism require further confirmation.

## 1. Introduction

*Litopenaeus vannamei* is one of the most widely cultured crustacean species worldwide and is a major contributor to marine aquaculture production and international seafood trade, particularly in China [1,2]. The intensification of shrimp farming has improved production efficiency but has also increased the exposure of cultured shrimp to multiple biotic and abiotic stressors, including pathogen infection, environmental fluctuation, high stocking density, and nutritional imbalance [3,4]. These stressors can compromise immune competence, disturb redox homeostasis, impair digestive and absorptive functions, and ultimately reduce growth performance, feed utilization, and survival [4,5]. Therefore, nutritional strategies that support immune competence, antioxidant defense, digestive function, and intestinal homeostasis are increasingly important for the sustainable development of shrimp aquaculture.

Functional feed additives, including probiotics, prebiotics, plant-derived bioactive compounds, and immunostimulants, have been widely investigated as nutritional strategies for improving the health and disease resilience of *L. vannamei*. Cytosine–phosphate–guanine oligodeoxynucleotides (CpG ODNs) are synthetic DNA molecules containing unmethylated CpG motifs that mimic immunostimulatory signals associated with microbial DNA [6]. In vertebrates, CpG ODNs can activate pattern-recognition receptor (PRR)-mediated signaling pathways and stimulate both innate and adaptive immune responses [6]. In aquatic animals, CpG ODNs have been reported to enhance pathogen resistance, increase immune- and antioxidant-related enzyme activities, regulate immune-associated gene expression, and improve stress tolerance [7,8]. Previous studies in crustaceans have shown that supplementation with individual CpG-based immunostimulants, such as CpG ODNs 1013 or pUC57-CpG, can enhance resistance to bacterial and other pathogenic infections and, in some cases, improve growth performance [8,9]. In the study on *Eriocheir sinensis*, dietary supplementation with multiple tandem CpG ODNs constructs at a fixed level showed that formulations containing CpG 1681, 2216, 2006, 2395, and 1651 exerted particularly significant growth-promoting and immune-enhancing effects [10]. Recent screening studies in shrimp have shown that, at the same dietary supplementation level, individual CpG ODNs sequences—including CpG-2216 (CpG-A), CpG-1681 (CpG-B), CpG-2006 (CpG-B), CpG-2395 (CpG-C), and CpG-21425 (CpG-P)—exert sequence-dependent effects on immune-related gene expression, antioxidant capacity, and intestinal microbiota composition [7,9]. Subsequently, different tandem CpG ODNs constructs were evaluated, and a preparation containing CpG 2216, 2006, 8954, 1826, and M362 showed particularly strong immunomodulatory effects, including enhanced immune and antioxidant responses and modulation of the intestinal microbial community [11]. A subsequent transcriptome and microRNAome study further demonstrated that dietary CpG ODNs supplementation affected multiple molecular processes associated with immune regulation, metabolism, autophagy, lysosomal function, and intracellular signaling [12]. Collectively, these studies have established the immunomodulatory potential of dietary CpG ODNss in shrimp. However, previous studies have largely focused on sequence or combination screening and mechanistic characterization at a fixed dietary supplementation level. Consequently, the dose–response relationship, effective dietary range, and optimal supplementation level of a defined multi-sequence CpG ODNs preparation remain poorly understood. Furthermore, it remains unclear whether different dietary levels of CpG ODNs induce coordinated or endpoint-specific responses in feed utilization, digestive function, immune status, disease resistance, intestinal microbiota, and host transcriptional regulation.

Based on this background, we hypothesized that graded dietary supplementation with a multi-sequence CpG ODNs preparation would induce dose-dependent, potentially non-linear responses in the physiological, immunological, microbial, and transcriptional status of *L. vannamei*. Therefore, this study aimed to evaluate the effects of dietary supplementation with graded levels of a multi-sequence CpG ODNs preparation on growth performance, immune status, digestive function, disease resistance, intestinal microbiota composition, and transcriptomic responses in *L. vannamei*. The research results are expected to provide a theoretical basis for the development of green, safe and efficient functional feed additives, and offer new insights for reducing the reliance on antibiotics and chemical therapies in aquaculture.

## 2. Materials and Methods

### 2.1. Experimental Diets

**Figure 1:**
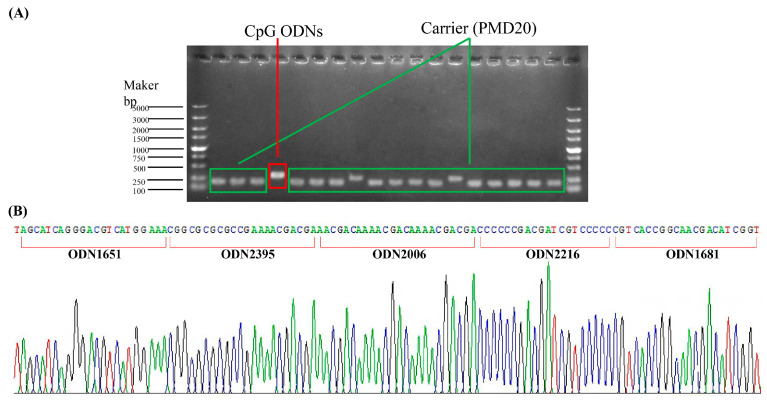
**Description of tandem ODNs sequence.** (**A**) Agarose gel electrophoresis. (**B**) Gene sequence detection. Sanger sequencing chromatogram confirming the assembly of ODN161, ODN2395, ODN2306, ODN2006, and ODN2186. The colored peaks represent the four nucleotide bases: A (green), T (red), C (blue), and G (black).

The CpG ODNs used in this study was designed as a composite CpG-rich oligodeoxynucleotide rather than as a de novo optimized sequence. Briefly, CpG motif-containing regions from five previously reported immunostimulatory CpG ODNss, namely ODN1651, ODN2395, ODN2006, ODN2216, and ODN1681, were tandemly arranged to generate one CpG-rich construct [11,12]. The final sequence of the composite CpG ODNs was 5′TAGCATCAGGGACGTCATGGAAACGGCGCGCGCCGAAAACGACGAAACGACAAAACGACAAAACGACGACCCCCCCGACGATCGTCCCCCCGTCACCGGCAACGACATCGGT3′. The boundaries of the five ODN-derived regions are indicated in Figure 1. Positive colonies were screened by PCR and agarose gel electrophoresis and subsequently submitted to Tsingke Biotechnology Co., Ltd. (Beijing, China). for sequencing verification. Colonies with correct sequencing results were selected for expanded culture. The synthesized CpG ODNs was prepared according to the instructions of a commercial kit purchased from Dongsheng Biological Co., Ltd. (Taixing, China). Briefly, the CpG ODNs preparation was boiled in water for 15 min and immediately cooled in an ice bath for 10 min to obtain a heat-treated CpG ODNs preparation. Its concentration was then determined, and the preparation was incorporated into the mixed feed at the levels shown in Table 1.

Fish meal, soybean meal, peanut meal, and corn gluten meal were used as the main protein sources, whereas corn oil, soybean lecithin, and fish oil were used as the main lipid sources. Based on previous studies on dietary CpG ODNs supplementation in *Eriocheir sinensis* [10] and *L. vannamei* [13], six isonitrogenous and isolipidic diets were formulated by supplementing the basal diet with different levels of CpG ODN. The dietary CpG ODN levels were set at 0, 0.1, 0.4, 1.6, 6.4, and 25.6 mg kg^−1^ feed, with the supplementation levels from 0.1 to 25.6 mg kg^−1^ arranged in a proportional gradient. The corresponding diets were designated C0, C0.1, C0.4, C1.6, C6.4, and C25.6, respectively, with C0 serving as the control. The formulation and nutrient composition of the experimental diets are presented in Table 1.

All dry ingredients were ground to pass through an 80-mesh sieve, accurately weighed, and thoroughly mixed using a laboratory mixer (M-256, South China University of Technology, Guangzhou, China) to ensure homogeneity. The mixed ingredients were then pelleted into 1.0 and 1.5 mm diameter pellets using a twin-screw extruder (F-26, South China University of Technology, Guangzhou, China). The pellets were dried in an oven at 60 °C for 30 min and then air-dried at room temperature until the moisture content reached approximately 10%. Finally, the diets were sealed in polyethylene zip lock bags and stored at −20 °C until use.

### 2.2. Experimental Design and Management

A total of 540 healthy *L. vannamei* with an initial body weight of 0.30 ± 0.02 g were randomly selected and assigned to six dietary treatment groups: C0 (control, 0 mg kg^−1^ CpG ODNs), C0.1 (0.1 mg kg^−1^ CpG ODNs), C0.4 (0.4 mg kg^−1^ CpG ODNs), C1.6 (1.6 mg kg^−1^ CpG ODNs), C6.4 (6.4 mg kg^−1^ CpG ODNs), and C25.6 (25.6 mg kg^−1^ CpG ODNs) groups. Each treatment group included three replicates, with 30 shrimp per replicate. The shrimp were reared in 18 tanks with a volume of 0.3 m^3^ each. Each dietary treatment comprised three physically independent tanks, and the tank was considered the experimental unit. During the 8-week feeding trial, shrimp were fed four times daily at 07:00, 11:00, 17:00, and 21:00. The daily feeding amount was maintained at approximately 3% of body weight. Forty minutes after feeding, uneaten feed was collected, air-dried, and weighed to calculate feed intake. During the experimental period, the water temperature was maintained at 28–30 °C, salinity at 28–30, and dissolved oxygen at ≥6.8 mg L^−1^. Approximately one-third of the rearing water in each tank was renewed daily to maintain water quality.

### 2.3. Sample Collection

The animal study protocol was approved by the Animal Ethics Committee of Guangdong Ocean University (protocol code GDOU-IACUC-2021-A3321; approval date: 15 March 2021). All procedures complied with institutional guidelines for the care and use of aquatic animals. At the end of the feeding trial, all shrimp in each tank were counted and weighed to calculate the survival rate (SR), final body weight (FBW), weight gain rate (WGR), specific growth rate (SGR), and feed conversion ratio (FCR). Each dietary treatment consisted of three independent tanks, which were the biological replicates and experimental units. Hemolymph was collected from each shrimp using a 1 mL sterile syringe. Hemolymph samples from three shrimp within the same tank were pooled to generate one tank-level composite sample. The shrimp used for pooling were considered subsamples from the same experimental unit and were not treated as additional biological replicates. The pooled hemolymph samples were kept at 4 °C for 12 h to allow clotting. Subsequently, the samples were centrifuged at 4000× *g* for 15 min at 4 °C, and the serum supernatant was collected and stored at −80 °C until subsequent analysis of serum biochemical parameters. In addition, hemocytes from six shrimp within each tank were pooled to generate two sample per tank. Hemolymph was collected as described above and centrifuged at 3000× *g* for 5 min at 4 °C to isolate hemocytes. Two hemocyte samples were immediately frozen in liquid nitrogen and stored at −80 °C until transcriptome sequencing and quantitative real-time PCR (qPCR) analysis. The intestinal samples were pooled into two samples per tank of six shrimp, with three intestines per composite sample, and immediately stored at −80 °C. One sample was used for the determination of intestinal digestive enzyme activities, and the other was used for intestinal microbiota profiling.

### 2.4. Pathogen Challenge Tests

After sampling, shrimp from each treatment group were pooled, and 20 individuals were randomly selected for the *Vibrio parahaemolyticus* and WSSV challenge tests, respectively. The preparation of *V. parahaemolyticus* and WSSV followed the procedures described in our previous study [14]. Half of the shrimp were intramuscularly injected with 50 μL of *V. parahaemolyticus* at a dose of 1.0 × 10^6^ CFU g^−1^ shrimp, while the remaining individuals were injected with 50 μL of WSSV at a dose of 1.0 × 10^5^ copies g^−1^ shrimp. Cumulative mortality was recorded at 4 h intervals for survival analysis.

### 2.5. Determination of Serum and Intestinal Biochemical Parameters

The activities of lysozyme (LZM), acid phosphatase (ACP), alkaline phosphatase (AKP), superoxide dismutase (SOD), phenoloxidase (PO), Aspartate aminotransferase (AST), and Alanine aminotransferase (ALT), as well as malondialdehyde (MDA) content, were measured in serum to evaluate non-specific immune and antioxidant parameters. Intestinal digestive enzyme activities, including amylase, trypsin, and lipase, were quantified as indicators of digestive function. All biochemical parameters were analysed using commercial assay kits obtained from Nanjing Jiancheng Bioengineering Institute (Nanjing, China), following the manufacturer’s instructions. Specifically, the activities of LZM, ACP, AKP, SOD, PO, CAT, AST, ALT, trypsin, amylase, and lipase, as well as MDA content, were measured using commercial colorimetric assay kits (A050-1-1, A060-1-1, A001-3-2, A136-1-1, A007-1-1, A059-1-1, A080-5-1, C009-2-1, C010-2-1, C016-1-1, H305-1-2, and A003-1-2, respectively) according to the manufacturer’s instructions. These assays were based on the formation of chromogenic products through specific enzymatic reactions, and enzyme activities were quantified by measuring absorbance at the appropriate wavelengths.

### 2.6. Sequencing and Analysis of Intestinal Microbiota

Intestinal samples stored at −80 °C were thawed, and bacterial DNA was extracted using a Magen’s HiPure Soil DNA Kit (Nanjing, China) according to the manufacturer’s instructions (*n* = 3).

The extracted intestinal bacterial samples were amplified, and the Illumina MiSeq platform was employed for the analysis of 16S rDNA. Detailed experimental procedures are outlined in the Supplementary Material (Text S1). The sequencing data have been deposited in the NCBI GenBank database under accession number PRJNA8234264.

### 2.7. Transcriptome Sequencing Analysis

Hemocyte samples stored at −80 °C were thawed on ice, and total RNA was extracted using a tissue RNA extraction kit (TransGen Biotech, Beijing, China) according to the manufacturer’s instructions. Three biological replicates were analyzed for each treatment (*n* = 3). The RNA samples were subsequently sent to Gene Denovo Biotechnology Co., Ltd. (Guangzhou, China) for library preparation and sequencing on the Illumina HiSeq 2000 platform. Further details of the RNA sequencing procedures are provided in Supplementary Material Text S2. The raw sequencing data have been deposited in the NCBI BioProject database under accession number PRJNA816062 for *L. vannamei*. To validate the RNA-seq results, 12 differentially expressed genes (DEGs) were selected from the transcriptomic dataset for quantitative real-time PCR (qPCR) analysis. cDNA was synthesized from the same RNA samples used for RNA sequencing using the PrimeScript™ RT Reagent Kit with gDNA Eraser (Beijing, China) according to the manufacturer’s instructions. Gene-specific primers were designed using Primer Premier 5.0 and are listed in Table S1. The relative expression levels of the selected genes were determined by qPCR. Further details of the qPCR procedures are provided in Supplementary Material Text S3.

### 2.8. Calculations and Statistical Analysis

Statistical analyses were performed using SPSS version 22.0 (SPSS, Armonk, NY, USA). Data were analyzed by one-way analysis of variance (ANOVA), followed by Duncan’s multiple range test to determine significant differences among treatment groups. Survival data after pathogen challenge were analyzed using Kaplan–Meier survival curves and compared using the log-rank test. RNA-seq DEGs was analyzed using DESeq2 package (version 1.40.2). All results are presented as the mean ± standard deviation (SD). Differences were considered statistically significant at *p* < 0.05. The SR, FBW, WGR, SGR, and FCR were used as growth performance indicators and were calculated according to the following equations:

SR (%) = final shrimp number/initial shrimp number × 100;

FBW = final body weight − initial body weight

WGR (%) = (final body weight − initial body weight)/initial body weight × 100;

SGR (%/day) = [ln (final body weight) − ln (initial body weight)]/days of feeding trial × 100;

FCR = weight of dry diet fed/wet weight gain.

## 3. Results

### 3.1. Growth Performance

**Figure 2:**
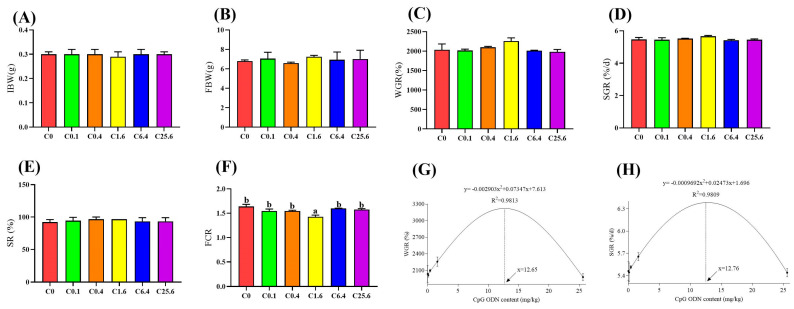
**Effects of dietary CpG ODNs supplementation on growth performance in *L. vannamei*.** (**A**) IBW, initial body weight; (**B**) FBW, final body weight; (**C**) WGR, weight gain rate; (**D**) SR, survival rate; (**E**) SGR, specific growth rate; (**F**) FCR, feed conversion ratio; (**G**) Regression curve of WGR against dietary CpG ODNs supplementation level; (**H**) Regression curve of SGR against dietary CpG ODNs supplementation level. Different superscript letters indicate significant differences among groups (*p* < 0.05), whereas values without superscript letters are not significantly different (*p* ≥ 0.05). The same notation is used in the subsequent figures and tables.

As shown in Figure 2, compared with the control group, the FCR was significantly lower in the C1.6 group, whereas no significant differences were observed among the other CpG ODNs-supplemented groups. In addition, IBW, FBW, WGR, SR, and SGR did not differ significantly among treatments. Based on regression analyses of WGR and SGR, the optimal dietary CpG ODNs supplementation levels were estimated to be 12.65 and 12.75 mg kg^−1^, respectively.

### 3.2. Pathogen Challenge Tests of L. vannamei

**Figure 3:**
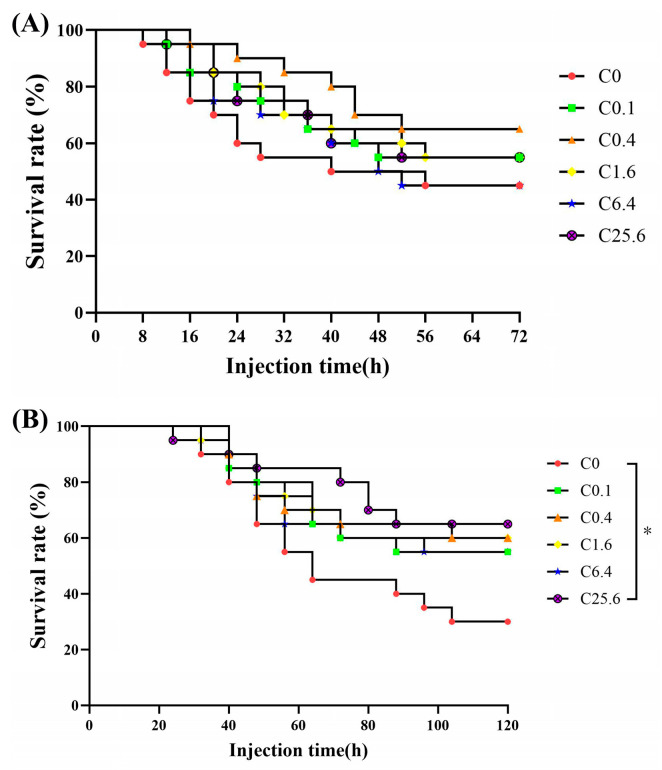
**Survival rates of *L. vannamei* after pathogen challenge.** (**A**) Survival rate of *V. parahaemolyticus* infection. (**B**) Survival rate of WSSV infection. The * was used to indicate significant differences (*p* < 0.05).

As shown in Figure 3, the SR of shrimp in all groups gradually decreased over time after pathogen challenge. Dietary CpG ODNs supplementation tended to increase the survival rate of *L. vannamei* after *V*. *parahaemolyticus* challenge. However, at 72 h post-challenge, no significant differences in SR were observed among the treatment groups (Figure 3A). At 120 h after WSSV injection, the SR of the C25.6 group was significantly higher than that of the control group (Figure 3B). No significant differences were detected among the other treatment groups.

### 3.3. Serum Biochemical Index Analysis

As shown in Table 2, compared with the control group, LZM activity was significantly increased in the C6.4 and C25.6 groups, while AKP activity was significantly higher in the C25.6 group. No significant differences in LZM or AKP activity were observed in the other CpG ODNs-supplemented groups. Compared with the control group, dietary CpG ODNs supplementation significantly increased serum ACP activity in *L. vannamei*, with the highest ACP activity observed in the C6.4 group. Except for SOD activity in the C0.1 group and CAT activity in the C0.1 and C0.4 groups, serum SOD, PO, and CAT activities were significantly higher in the CpG ODNs-supplemented groups than in the control group. The highest SOD and CAT activities were observed in the C6.4 group, whereas the highest PO activity was detected in the C1.6 group. Compared with the control group, AST activity was significantly higher in the C0.1 group, whereas no significant differences were observed in the other treatment groups. For ALT activity, the C0.4 group showed a significant increase compared with the control group, whereas the C1.6, C6.4, and C25.6 groups showed significant decreases. No significant difference in ALT activity was observed between the C0.1 and control groups. Compared with the control group, dietary CpG ODNs supplementation significantly reduced serum MDA content in all supplemented groups except the C0.1 group.

### 3.4. Digestive Enzyme Activities in the Intestine

As shown in Table 3, compared with the control group, intestinal trypsin, amylase, and lipase activities were significantly increased in the C1.6, C6.4, and C25.6 groups. Among these treatments, the highest trypsin and lipase activities were observed in the C1.6 group, whereas the highest amylase activity was detected in the C6.4 group. No significant differences in digestive enzyme activities were observed in the C0.1 group compared with the control group. In the C0.4 group, trypsin and amylase activities were significantly increased, whereas lipase activity showed no significant difference. In addition, TP content did not differ significantly among the treatment groups.

### 3.5. Intestinal Microbiota Community

#### 3.5.1. Intestinal Microbiota Diversity and Richness

**Figure 4:**
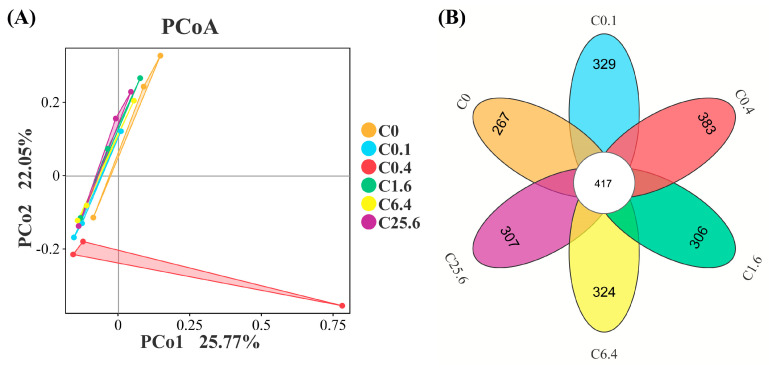
**Principal coordinate analysis and Venn diagram.** (**A**) PcoA analysis of intestinal flora. (**B**) Comparison of OTU Venn diagrams.

As shown in Figure 4A, principal coordinate analysis (PCoA) based on beta diversity indicated that dietary supplementation with different levels of CpG ODNs affected the intestinal microbial community, with clear differences observed among groups. The first principal coordinate (PC1) explained 25.77% of the total variation, whereas the second principal coordinate (PC2) explained 22.05%. Venn diagram analysis showed that the C0, C0.1, C0.4, C1.6, C6.4, and C25.6 groups contained 267, 329, 383, 306, 324, and 307 unique OTUs, respectively (Figure 4B). Alpha-diversity analysis showed that, compared with the control group, dietary CpG ODNs supplementation significantly increased the Sobs, ACE, and Chao1 indices of the intestinal microbiota in *L. vannamei* (Table 4). No significant differences in the Shannon or Simpson indices were observed among the treatment groups.

#### 3.5.2. Composition of Intestinal Microbiota

**Figure 5:**
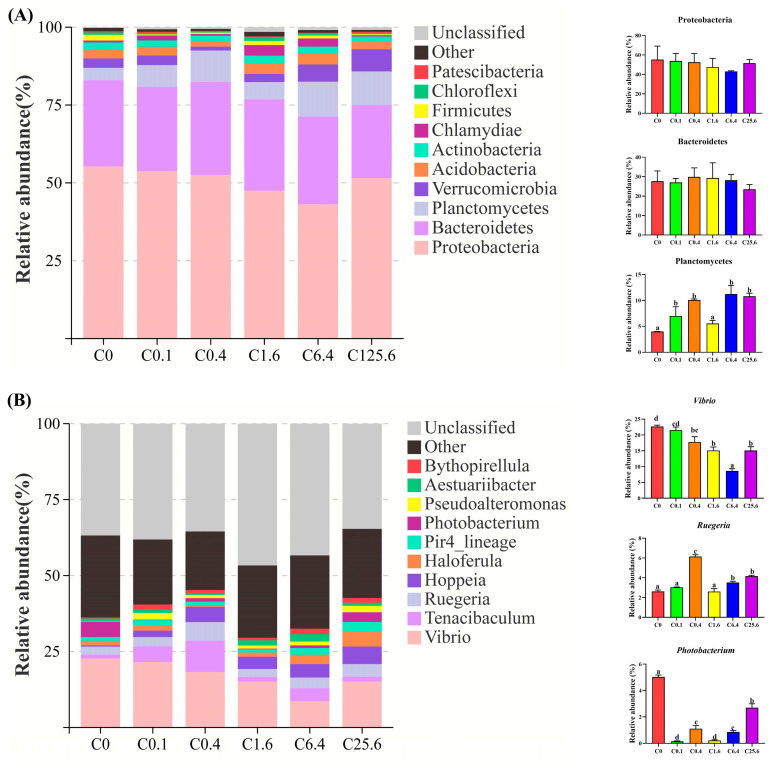
**Structure and composition of the intestinal microbial communities in *L. vannamei* fed diets containing different levels of CpG ODNs.** (**A**) Composition of the intestinal microbiota at the phylum level. (**B**) Composition of the intestinal microbiota at the genus level. Different superscript letters indicate significant differences among groups (*p* < 0.05), whereas values without superscript letters are not significantly different (*p* ≥ 0.05).

As shown in Figure 5A, the intestinal microbiota was dominated by Proteobacteria, Bacteroidetes, Planctomycetes, and Verrucomicrobia at the phylum level. Compared with the control group, dietary CpG ODNs supplementation did not significantly affect the relative abundances of Proteobacteria or Bacteroidetes. The relative abundance of Planctomycetes was significantly higher in the C0.1, C0.4, C6.4, and C25.6 groups than in the control group, whereas no significant difference was observed between the C1.6 and control groups. As shown in Figure 5B, the intestinal microbiota was dominated by *Vibrio*, *Tenacibaculum*, *Ruegeria*, and *Hoppeia* at the genus level. Compared with the control group, the relative abundances of *Vibrio* and *Photobacterium* were significantly reduced in the CpG ODNs-supplemented groups, except that *Vibrio* abundance in the C0.1 group did not differ significantly from that in the control group. The relative abundance of *Ruegeria* was significantly higher in the C0.4, C6.4, and C25.6 groups than in the control group, whereas no significant differences were observed between the C0.1, or C1.6 groups and the control group.

### 3.6. Transcriptome Sequencing Analysis

#### 3.6.1. Assembly and Sequence Alignment Analysis

The raw data of this study has been deposited in the SRA database with the accession number PRJNA816062. As shown in Table S1, sequencing was performed using the Illumina platform. After redundant and low-quality sequences were removed from the original 51,303,154 bp dataset, an average of 51,130,138 bp of clean data was obtained. The mean GC content of each group was greater than 49.80%, with Q20 and Q30 values exceeding 97.17% and 92.28%, respectively. The mean overall mapping rate was greater than 88.65%; the proportion of reads mapped to exon regions exceeded 88.05%, whereas that mapped to intron regions was below 7.27%. The assembly results indicate that the sequencing is of good quality and can be used for transcriptome analysis.

#### 3.6.2. Analysis of DEGs

**Figure 6:**
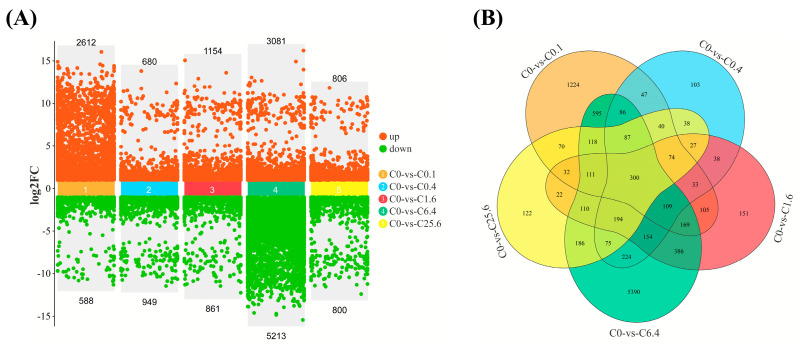
**Analysis of DEGs.** (**A**) DEGs between *L. vannamei* containing CpG ODNs supplementation group and control groups. (**B**) Venn diagram of DEGs.

As shown in Figure 6A, DEGs were identified in the five comparison groups. In the C0 vs. C0.1 comparison group, 3200 DEGs were detected, including 2612 upregulated and 588 downregulated genes. In the C0 vs. C0.4 comparison group, 1629 DEGs were identified, including 680 upregulated and 949 downregulated genes. In the C0 vs. C1.6 comparison group, 2015 DEGs were detected, of which 1154 were upregulated and 861 were downregulated. The C0 vs. C6.4 comparison group showed the highest number of DEGs, with a total of 8294 DEGs, including 3081 upregulated and 5213 downregulated genes. In the C0 vs. C25.6 comparison group, 1606 DEGs were identified, including 806 upregulated and 800 downregulated genes. As shown in Figure 6B, 300 DEGs were shared among all comparison groups.

#### 3.6.3. GO Enrichment Analysis of DEGs

**Figure 7:**
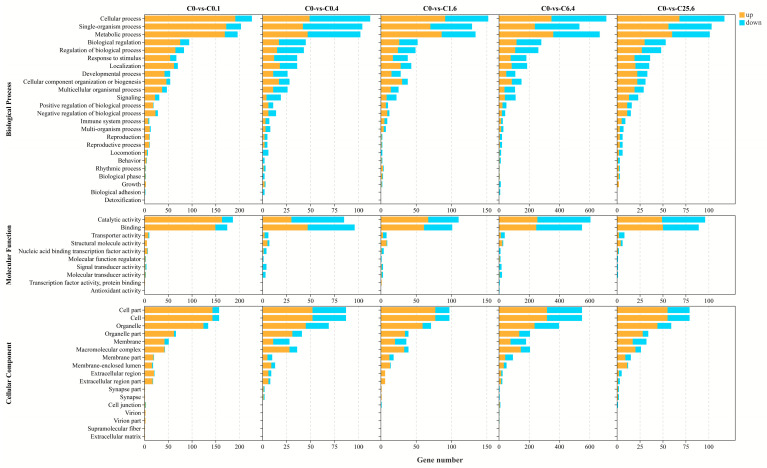
**GO enrichment analysis of DEGs**. GO enrichment analysis of DEGs. Three main GO categories: cellular components, molecular functions and biological processes. x-axis indicates the number of DEGs and y-axis indicates GO categories and subcategories.

As shown in Figure 7, GO enrichment analysis revealed that the DEGs between the CpG ODNs supplementation and control groups were assigned to 51 level-2 GO subcategories. Among these, 24 subcategories were associated with biological processes, mainly including cellular processes, metabolic processes, single-organism processes, biological regulation, signaling, and localization. Seventeen subcategories were assigned to cellular components, including macromolecular complexes, extracellular regions, membrane-enclosed lumens, and supramolecular fibers. The remaining 10 subcategories belonged to molecular functions, such as catalytic activity, transcription factor activity, signal transducer activity, structural molecule activity, and molecular transducer activity.

#### 3.6.4. KEGG Enrichment Analysis of DEGs

**Figure 8:**
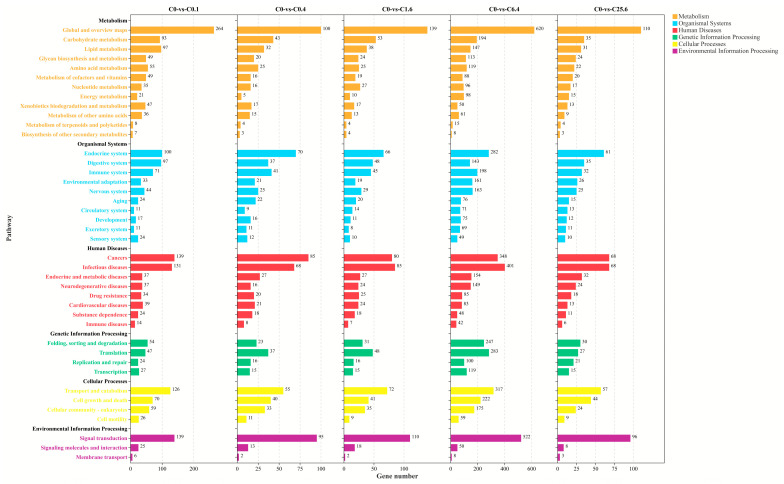
**KEGG enrichment analysis of DEGs**. KEGG enrichment analysis of DEGs. Highly expressed biological pathways in the transcriptome were retrieved from the KEGG database. DEGs were assigned to six specific KEGG pathways, including organismal systems, metabolism, genetic information processing and environmental information processing, cellular processes and human diseases.

**Figure 9:**
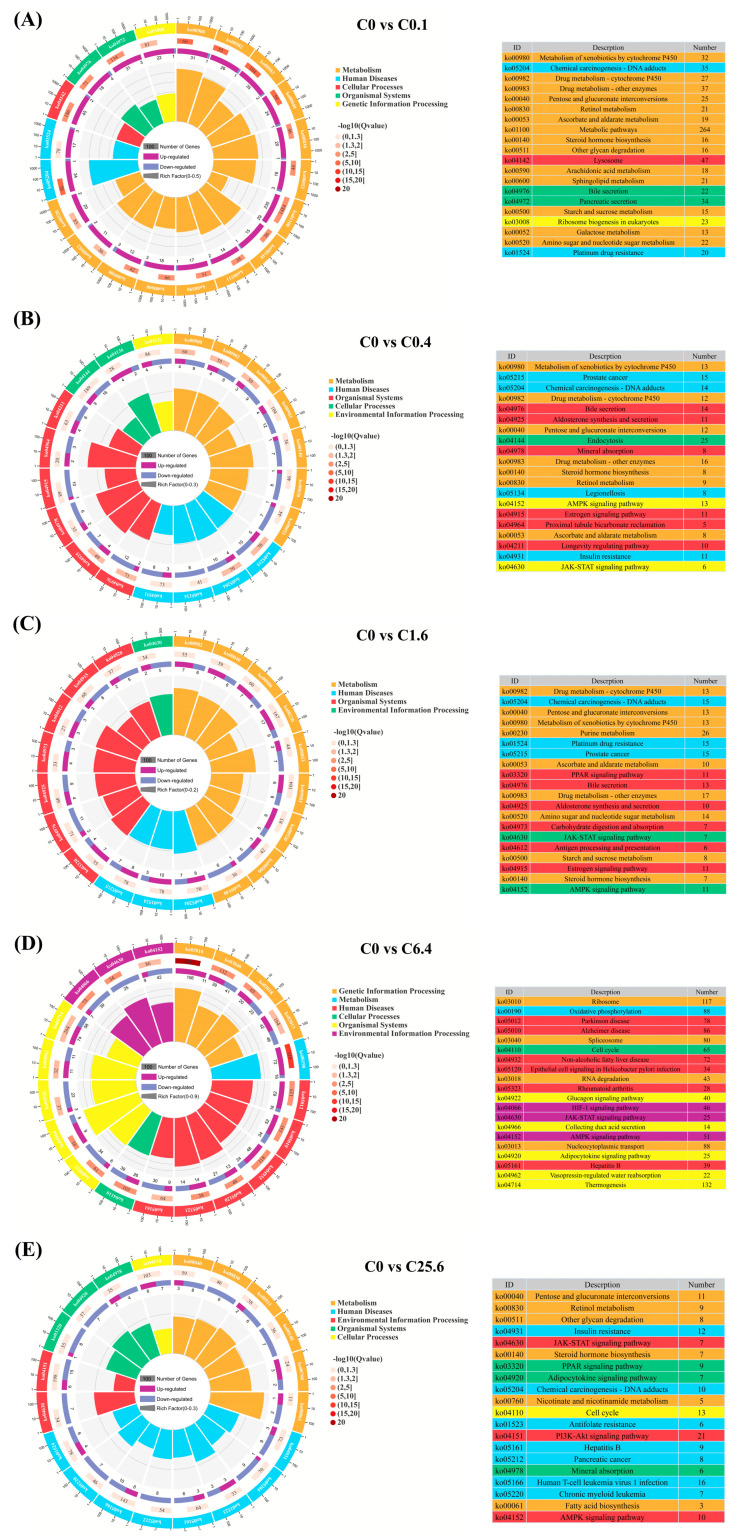
**Top 20 pathways for KEGG enrichment analysis.** (**A**) stand for C0-vs-C0.1 group, (**B**) stand for C0-vs-C0.4 group, (**C**) stand for C0-vs-C1.6 group, (**D**) stand for C0-vs-C6.4 group, (**E**) stand for C0-vs-C25.6 group.

To identify the biological pathways potentially affected by dietary CpG ODNs supplementation, the DEGs were mapped to the KEGG database for pathway enrichment analysis. As shown in Figure 8, the DEGs identified between each CpG ODNs-supplemented group and the control groups were distributed across 41 KEGG subcategories, which were assigned to six major KEGG categories: metabolism (12), organismal systems (10), human diseases (8), genetic information processing (4), cellular processes (4), and environmental information processing (3). A relatively large number of DEGs were enriched in pathways associated with global and overview maps, the endocrine system, cancers, infectious diseases, folding, sorting and degradation, translation, transport and catabolism, and signal transduction. However, the top 20 enriched pathways differed among the CpG ODNs-supplemented groups. As shown in Figure 9, only the top 20 enriched pathways in the comparison group between C6.4 and C0 covered all six KEGG categories. In the C0 vs. C0.1 comparison group, 15 of the top 20 enriched pathways were related to metabolism (Figure 9A), with most DEGs enriched in metabolic pathways (ko01100), lysosome (ko04142), drug metabolism–other enzymes (ko00983), and pancreatic secretion (ko04972). Compared with the control group, DEGs in the C0 vs. C0.4 comparison group were mainly enriched in cytokinesis/endocytosis-related processes (ko04144), the AMPK signaling pathway (ko04152), aldosterone synthesis and secretion (ko04925), and bile secretion (ko04976; Figure 9B). In the C0 vs. C1.6 comparison group, most DEGs were associated with drug metabolism–other enzymes (ko00983), purine metabolism (ko00230), amino sugar and nucleotide sugar metabolism (ko00520), and chemical carcinogenesis (ko05204; Figure 9C). In the C0 vs. C6.4 comparison group, the ribosome pathway (ko03010), oxidative phosphorylation (ko00190), and thermogenesis were the three pathways containing the highest numbers of DEGs compared with the control group (Figure 9D). As shown in Figure 9E, 10 of the top 20 enriched pathways in the C0 vs. C25.6 comparison group were associated with human diseases, two with organismal systems, and four with metabolism. The DEGs in this group were mainly enriched in the PI3K-Akt signaling pathway (ko04151), cell cycle (ko04110), and insulin resistance (ko04931). Notably, compared with the control group, the C0.4, C1.6, C6.4, and C25.6 groups shared significant enrichment in several immune-related pathways, including the AMPK signaling pathway, and JAK-STAT signaling pathway.

#### 3.6.5. Validation of qPCR

**Figure 10:**
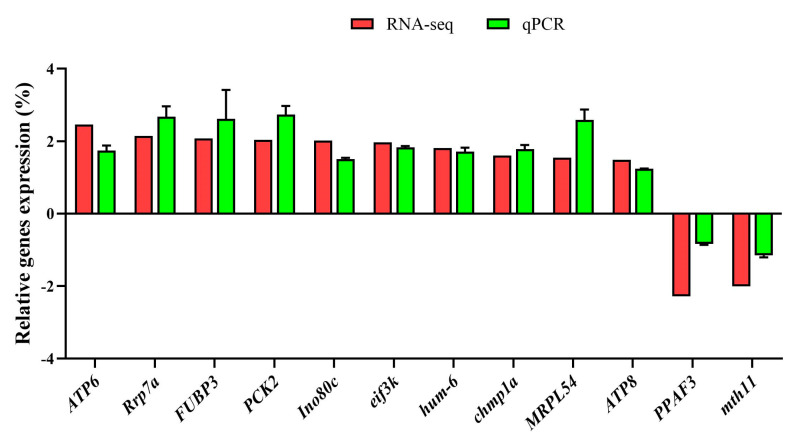
**Validation of the associated DEGs by qPCR.** Gene expression assays were performed in triplicate for each sample. Expression values were normalized to those of EF1α using the Livak and Schmittgen method (2^−ΔΔCt^), and data are the mean ± SD of triplicate assays.

Twelve DEGs, including 10 upregulated and 2 downregulated genes, were randomly selected for validation. The qPCR results showed expression patterns consistent with the transcriptome sequencing data (Figure 10).

## 4. Discussion

In the present study, dietary supplementation with CpG ODNs at levels exceeding 0.1 mg kg^−1^ did not significantly improve the growth performance of *L. vannamei*. Nevertheless, regression analyses based on WGR and SGR indicated that the predicted optimal supplementation levels were 12.65 and 12.75 mg kg^−1^, respectively, suggesting a potential dose-dependent trend within the tested range. These results differ from those reported in *Eriocheir sinensis*, in which dietary supplementation with 40 or 100 mg kg^−1^ CpG ODNs significantly enhanced growth performance [10]. This discrepancy may be attributed to differences in species, developmental stage, basal diet composition, culture conditions, experimental duration, and the sensitivity and physiological responses of shrimp and crab species to immunostimulants. Digestive enzyme activities reflect the capacity of shrimp to hydrolyze and utilize proteins, carbohydrates, and lipids [15]. Increased activities of these enzymes may contribute to improved intestinal function and digestive capacity [16]. In this study, trypsin, amylase, and lipase activities were significantly increased in shrimp fed diets supplemented with 1.6, 6.4, and 25.6 mg kg^−1^ CpG ODNs, with the highest trypsin and lipase activities observed in the 1.6 mg kg^−1^ supplementation group. These results suggest that the reduced FCR observed at this dose may be associated with enhanced intestinal digestive capacity. Nevertheless, dietary supplementation with CpG ODNs at levels exceeding 0.1 mg kg^−1^ did not significantly improve the overall growth performance of *L. vannamei* in the present study. This may be because the increase in digestive enzyme activity was not sufficient to translate into a stable growth advantage.

Crustaceans, such as shrimp and crabs, lack a classical adaptive immune system and do not produce specific antibodies [10,17,18]. Immune-related enzymes, including LZM, AKP, ACP, and PO, are commonly used as indicators to evaluate the non-specific immune status of crustaceans [10,17,18]. Studies on *Eriocheir sinensis*, *L. vannamei*, and *Penaeus monodon* have shown that appropriate levels of immunostimulants can enhance the activities of immune-related enzymes, such as LZM, AKP, and ACP, thereby improving non-specific immune responses in cultured animals [10,17,18]. Consistently, the present study showed that dietary supplementation with CpG ODNs 25.6 mg kg^−1^ significantly increased the activities of LZM, AKP, ACP, and PO and significantly improved the post-challenge survival after WSSV challenge. These results suggest that appropriate dietary levels of CpG ODNs can enhance non-specific immune responses in *L. vannamei*. In addition, antioxidant enzymes such as SOD and CAT, together with MDA content, are widely used as indicators of oxidative stress and antioxidant defense capacity. In the present study, dietary supplementation with CpG ODNs at levels above 1.6 mg kg^−1^ significantly increased the activities of SOD and CAT, while reducing MDA content. Consistent with these findings, dietary supplementation with 50 mg kg^−1^ CpG ODNs has been reported to significantly enhance the activities and mRNA expression levels of antioxidant enzymes, including SOD, CAT, and GPX, in the hepatopancreas and intestine of *L. vannamei*. It also reduced MDA content, thereby improving antioxidant capacity [11,13]. These findings suggest that appropriate dietary levels of CpG ODNs can enhance the antioxidant capacity of *L. vannamei*. Notably, dietary CpG ODNs supplementation resulted in numerically higher survival in *L. vannamei* following *V. parahaemolyticus* challenge; however, the differences among treatment groups were not statistically significant. In contrast, shrimp in the C25.6 group showed a significantly higher survival rate after white WSSV challenge. These results indicate that CpG ODNs did not confer broad protection against both viral and bacterial pathogens under the present experimental conditions, but instead showed a pathogen-specific. Although CpG motifs are present in both viral and bacterial DNA, the final protective outcome induced by CpG ODNs cannot be explained solely by the presence of CpG-containing DNA. A possible explanation may be related to differences in pathogen-associated molecular patterns (PAMPs), pattern-recognition receptor (PRR) activation, and downstream effector requirements between viral and bacterial infections in crustaceans. CpG ODNs mimics unmethylated CpG-rich microbial DNA and may preferentially prime host responses associated with nucleic-acid recognition and systemic hemocyte-mediated antiviral defense. WSSV is a systemic double-stranded DNA virus that infects multiple shrimp tissues and may be more susceptible to CpG ODNs-induced antiviral immune priming, including hemocyte-mediated responses and conserved antiviral signaling pathways [19,20]. In contrast, *V. parahaemolyticus* is a rapidly proliferating bacterial pathogen whose pathogenicity involves colonization, secretion systems, virulence-factor production, and toxin-mediated tissue damage [21,22]. Effective antibacterial protection in shrimp may therefore require coordinated activation of antibacterial PRRs and effector mechanisms, including lectin-like recognition, prophenoloxidase activation, phagocytosis, antimicrobial peptides, and mucosal barrier responses. Although serum immune markers such as LZM, ACP, and PO were enhanced by CpG ODNs supplementation, these generalized immune responses may not have been sufficient to overcome the acute pathogenic pressure caused by the *V. parahaemolyticus* challenge. Therefore, the absence of significant protection against *V. parahaemolyticus* should not be interpreted as evidence that CpG ODNs lacks immunostimulatory activity. Rather, it suggests that the protective efficacy of CpG ODNs may depend on the type of pathogen, the dominant PAMP–PRR recognition axis, the infection route, and the immune effector mechanisms required for pathogen clearance. Future studies should evaluate multiple bacterial challenge doses, oral or immersion infection models, bacterial load dynamics, tissue damage, and antibacterial immune pathways to determine whether CpG ODNs can improve resistance to bacterial pathogens under different infection scenarios.

The intestinal microbiota plays an important role in regulating host metabolism, immune function, and disease resistance, thereby influencing the health status of *L. vannamei* [23,24]. Previous studies have shown that disease outbreaks in *L. vannamei* are often associated with reduced intestinal microbial diversity, which may further compromise host health [25]. In this study, dietary supplementation with CpG ODNs significantly increased the Chao1 and ACE indices of the intestinal microbiota. These results suggest that CpG ODNs may improve intestinal microbial richness and potentially contribute to enhanced disease resistance in *L. vannamei*. Previous studies have shown that Proteobacteria, Bacteroidetes, Planctomycetes, and Verrucomicrobia are the dominant bacterial phyla in the intestinal microbiota of *L. vannamei* [26,27]. Consistent with these findings, the dominant intestinal bacterial phyla observed in *L. vannamei* fed diets supplemented with CpG ODNs were similar to those reported in previous studies. *Vibrio* and *Photobacterium* are common opportunistic pathogens in shrimp and can adversely affect host health [28,29,30]. In contrast, several studies have suggested that the abundance of *Ruegeria* is positively associated with shrimp health and negatively associated with the abundance of pathogenic bacteria, such as *Vibrio* [1,22]. A similar pattern has also been reported in healthy fiddler crabs, in which *Ruegeria* was identified as one of the dominant intestinal bacterial genera [31,32]. In this study, dietary supplementation with CpG ODNs at levels exceeding 0.4 mg kg^−1^ significantly reduced the relative abundances of *Vibrio* and *Photobacterium*, whereas supplementation at 0.4, 6.4, and 25.6 mg kg^−1^ significantly increased the relative abundance of *Ruegeria*. These findings indicate that dietary supplementation with CpG ODNs at levels exceeding 0.4 mg kg^−1^ may improve the intestinal microbial composition of *L. vannamei*.

Transcriptomic analysis can be used to infer the potential functions of uncharacterized genes based on their expression patterns under specific experimental conditions, thereby providing insights into the roles of genes in related biological pathways [33,34,35]. In shrimp, transcriptome sequencing has been widely applied for functional gene annotation, screening of DEGs, and development of molecular markers [36,37]. In this study, regression analyses based on weight gain rate and specific growth rate showed that the predicted peak growth performance occurred at 12.65 and 12.75 mg kg^−1^, respectively. Both values were within the tested dietary interval between 6.4 and 25.6 mg kg^−1^. These results suggest that the growth-promoting effect of CpG ODNs is non-linear. Transcriptomic analysis further supported this conclusion, showing that 8294 DEGs were identified in the C6.4 group, whereas 1606 DEGs were identified in the C25.6 group. This finding indicates that the effect of CpG ODNs on gene expression also exhibited a non-linear pattern, which is consistent with the regression curve analysis of growth performance. Furthermore, KEGG pathway enrichment analysis showed that although the transcriptional responses induced by different dietary levels of CpG ODNs exhibited a certain degree of dose specificity, they also shared several common features. The DEGs in each comparison group were broadly distributed across pathways related to metabolism, cellular processes, environmental information processing, genetic information processing, organismal systems, and disease-related categories. These results suggest that dietary CpG ODNs supplementation may not act through a single immune pathway in penaeid shrimp, but rather modulate physiological status through multiple biological processes at different regulatory levels. Notably, compared with the control group, the C0.4, C1.6, C6.4, and C25.6 groups showed common significant enrichment in several pathways, including the AMPK signaling pathway and Jak-STAT signaling pathway. The JAK–STAT signaling pathway is closely associated with immune regulation and is involved in immune cell differentiation and inflammatory responses [38]. The AMPK signaling pathway may indirectly contribute to immune regulation by suppressing inflammation and promoting anti-inflammatory macrophage polarization [39,40]. These findings indicate that dietary supplementation with CpG ODNs at levels exceeding 0.4 mg kg^−1^ affected the AMPK signaling pathway and JAK-STAT signaling pathway, thereby potentially modulating immune regulatory capacity.

Several limitations of the present study should be acknowledged. The dietary CpG ODNs levels used in this study were designed to provide an initial broad-range evaluation of CpG ODNs supplementation in *L. vannamei*. However, no intermediate dietary CpG ODNs levels were included between 6.4 and 25.6 mg kg^−1^, which limits the resolution of the dose–response relationship in the higher supplementation range. Therefore, the beneficial effects observed at 25.6 mg kg^−1^ should be interpreted as indicating an effective level under the present experimental conditions, rather than as definitive evidence of the optimal supplementation level. The number of independent tanks per treatment was limited to three. Although multiple shrimp were sampled from each tank to improve the representativeness of tank-level measurements, the tank, rather than the individual shrimp, was the experimental unit. Therefore, the statistical power to detect small treatment effects may have been limited, particularly considering the large number of endpoints evaluated, including growth performance, digestive and immune-related enzyme activities, antioxidant indices, intestinal microbiota, WSSV challenge survival, and transcriptomic responses. In addition, the present feeding trial was conducted under highly controlled aquarium conditions and was limited to an 8-week observation period during the juvenile stage of *L. vannamei.* Therefore, the results may not fully reflect the effects of CpG ODNs supplementation throughout a complete commercial pond production cycle until market size. In particular, the long-term effects of CpG ODNs on growth performance, feed utilization, disease resistance, intestinal microbiota stability, and host physiological responses remain unclear. Moreover, because CpG ODNs is relatively expensive, the present study cannot determine whether its biological benefits are sufficient to justify its use as a practical feed additive under commercial farming conditions. Accordingly, the present findings should be interpreted as preliminary evidence of the potential beneficial effects of dietary CpG ODNs in *L. vannamei* under controlled experimental conditions. Further studies with narrower dose intervals, more independent tank replicates, prospectively determined sample sizes, longer feeding durations, and commercial pond-scale validation are needed to confirm the dose–response relationship, optimal supplementation level, long-term growth effects, economic cost-effectiveness, and the stability of intestinal microbiota throughout the production cycle until market size.

## 5. Conclusions

Dietary CpG ODNs supplementation did not significantly improve the growth performance of *L*. *vannamei*. However, supplementation with 25.6 mg kg^−1^ CpG ODNs enhanced resistance to WSSV challenge. Dietary supplementation with CpG ODNs at levels above 0.4 mg kg^−1^ significantly increased digestive and immune enzyme activities, improved intestinal microbiota composition, and induced beneficial immune-related transcriptomic responses.

## Figures and Tables

**Table 1:** **Ingredient and proximate composition of the experimental diets (% dry matter).**

Ingredient	C0	C0.1	C0.4	C1.6	C6.4	C25.6
Brown fish meal	20	20	20	20	20	20
Soybean meal	20	20	20	20	20	20
Shrimp head meal	4	4	4	4	4	4
Peanut meal	9	9	9	9	9	9
Corn gluten meal	10	10	10	10	10	10
Wheat meal	25	25	25	25	25	25
Fish oil	2	2	2	2	2	2
Corn oil	2	2	2	2	2	2
Soybean lecithin	0.5	0.5	0.5	0.5	0.5	0.5
^a^ Vitamin premix	0.2	0.2	0.2	0.2	0.2	0.2
^b^ Mineral premix	0.5	0.5	0.5	0.5	0.5	0.5
Choline chloride	0.5	0.5	0.5	0.5	0.5	0.5
Antioxidants	0.03	0.03	0.03	0.03	0.03	0.03
Lunar agent	0.1	0.1	0.1	0.1	0.1	0.1
Calcium phosphate	1.5	1.5	1.5	1.5	1.5	1.5
Vitamin C	0.05	0.05	0.05	0.05	0.05	0.05
Microcrystalline cellulose	4.62	4.62	4.62	4.62	4.62	4.62
CpG ODNs (mg kg^−1^)	0	0.1	0.4	1.6	6.4	25.6
Total	100	100	100	100	100	100
^c^ Crude protein	40.21	40.12	39.98	40.05	39.89	40.04
^c^ Crude lipid	7.72	8.01	7.82	7.91	8.20	7.76
^c^ Ash	12.53	12.42	13.15	12.34	13.08	12.67

Notes: ^a^ Vitamin premix (per kg diet): Vitamin B1, 25 mg; Vitamin B2, 45 mg; Vitamin B3, 60 mg; Vitamin B5, 200 mg; Vitamin B6, 20 mg; Vitamin B7, 1.20 mg; Vitamin B12, 0.1 mg; Inositol, 800 mg; Folic acid, 20 mg; Vitamin A, 32 mg; Vitamin E, 120 mg; Vitamin D3, 5 mg; Vitamin K3, 10 mg. ^b^ Mineral premix (per kg diet): Sodium fluoride, 2 mg; Potassium iodide, 0.8 mg; Cobalt chloride (%), 50 mg; Cupric sulphate, 10 mg; Ferrous sulphate, 80 mg; Zinc sulphate, 50 mg; Manganese sulphate, 60 mg; Magnesium sulphate, 1200 mg; Sodium chloride, 100 mg; Zeolite powder, 1447.2 mg. ^c^ Crude protein, crude lipid and ash contents were measured value.

**Table 2:** **Serum biochemical indicators of *L. vannamei*.**

Index	C0	C0.1	C0.4	C1.6	C6.4	C25.6
LZM (U/L)	4.43 ± 0.20 ^b^	3.87 ± 0.26 ^ab^	4.80 ± 0.16 ^b^	5.07 ± 0.09 ^bc^	5.70 ± 0.09 ^cd^	5.89 ± 0.12 ^d^
ACP (U/L)	4.77 ± 0.20 ^a^	6.74 ± 0.06 ^b^	7.66 ± 0.05 ^cd^	7.30 ± 0.10 ^c^	8.39 ± 0.04 ^e^	8.14 ± 0.10 ^de^
AKP (U/L)	14.20 ± 0.26 ^b^	11.59 ± 0.06 ^ab^	12.05 ± 0.18 ^ab^	15.02 ± 0.25 ^bc^	15.06 ± 0.29 ^bc^	15.91 ± 0.14 ^c^
SOD (U/mL)	82.94 ± 7.40 ^a^	98.34 ± 8.44 ^ab^	118.43 ± 7.00 ^bc^	138.40 ± 2.63 ^cd^	147.46 ± 16.56 ^bc^	126.70 ± 7.61 ^cd^
PO (U/mL)	12.67 ± 0.81 ^a^	21.09 ± 3.45 ^b^	19.39 ± 3.30 ^b^	26.68 ± 0.84 ^d^	22.27 ± 2.88 ^bc^	25.42 ± 1.89 ^cd^
CAT (U/mL)	7.35 ± 1.07 ^a^	7.48 ± 1.40 ^a^	9.18 ± 1.16 ^ab^	9.57 ± 1.14 ^bc^	11.41 ± 0.86 ^c^	9.67 ± 1.32 ^bc^
AST (U/L)	12.78 ± 0.23 ^ab^	15.51 ± 0.31 ^c^	14.85 ± 0.32 ^bc^	14.90 ± 0.02 ^bc^	15.11 ± 0.18 ^bc^	14.13 ± 0.04 ^b^
ALT (U/L)	7.08 ± 0.06 ^c^	6.85 ± 0.09 ^c^	7.52 ± 0.09 ^d^	6.40 ± 0.08 ^b^	5.97 ± 0.09 ^ab^	6.26 ± 0.03 ^ab^
MDA (nmol/mL)	10.68 ± 0.47 ^b^	10.03 ± 1.05 ^b^	6.93 ± 0.48 ^a^	6.43 ± 0.78 ^a^	7.01 ± 1.48 ^a^	8.00 ± 1.29 ^a^

Note: LZM, lysozyme; ACP, acid phosphatase; AKP, alkaline phosphatase; PO, phenoloxidase; SOD, superoxide dismutase; CAT, catalase; AST, Aspartate aminotransferase; ALT, Alanine aminotransferase; MDA, malondialdehyde. All data are presented as the mean ± SD (*n* = 3). Different superscript letters indicate significant differences among groups (*p* < 0.05), whereas values without superscript letters are not significantly different (*p* ≥ 0.05).

**Table 3:** **Intestinal digestive enzyme activities in *L. vannamei*.**

Index	C0	C0.1	C0.4	C1.6	C6.4	C25.6
TP (ng/mg tissue)	653.10 ± 45.87	610.09 ± 47.78	585.19 ± 52.17	606.43 ± 66.48	606.43 ± 70.65	625.40 ± 53.58
Lipase (U/mg pro)	532.25 ± 3.57 ^a^	544.56 ± 6.22 ^a^	557.92 ± 12.1 ^ab^	749.32 ± 6.93 ^c^	747.92 ± 4.24 ^c^	580.63 ± 7.22 ^b^
Trypsin (U/mg pro)	596.00 ± 3.74 ^a^	618.29 ± 17.32 ^a^	805.01 ± 18.2 ^b^	1085.04 ± 7.55 ^d^	1037.92 ± 23.1 ^d^	935.41 ± 23.63 ^c^
Amylase (U/mg pro)	174.27 ± 3.26 ^a^	181.67 ± 1.72 ^a^	255.54 ± 4.73 ^bc^	269.20 ± 9.99 ^bc^	274.49 ± 4.85 ^c^	246.03 ± 5.65 ^b^

Note: TP, total protein. All data are presented as the mean ± SD (*n* = 3). Different superscript letters indicate significant differences among groups (*p* < 0.05), whereas values without superscript letters are not significantly different (*p* ≥ 0.05).

**Table 4:** **Effects of dietary CpG ODNs on intestinal microbiota alpha-diversity indices in *L. vannamei*.**

Index	C0	C0.1	C0.4	C1.6	C6.4	C25.6
Sobs	1152.50 ± 74.25 ^a^	1697.50 ± 115.26 ^b^	1883.50 ± 20.51 ^b^	1646.00 ± 28.28 ^b^	1717.50 ± 89.80 ^b^	1702.00 ± 135.76 ^b^
Shannon	6.29 ± 0.42	6.76 ± 0.48	6.74 ± 0.66	7.28 ± 0.18	7.51 ± 0.37	7.23 ± 0.20
Simpson	0.95 ± 0.05	0.97 ± 0.01	0.96 ± 0.02	0.98 ± 0.01	0.98 ± 0.01	0.98 ± 0.00
Chao1	1979.86 ± 53.56 ^a^	2896.24 ± 267.17 ^b^	2870.14 ± 37.85 ^b^	2788.03 ± 142.53 ^b^	2836.78 ± 108.86 ^b^	2834.91 ± 213.35 ^b^
ACE	1847.44 ± 18.47 ^a^	2841.85 ± 269.56 ^b^	2895.25 ± 3.78 ^b^	2697.84 ± 136.57 ^b^	2724.18 ± 50.29 ^b^	2627.00 ± 382.61 ^b^

Note: All data are presented as the mean ± SD (*n* = 3). Different superscript letters indicate significant differences among groups (*p* < 0.05), whereas values without superscript letters are not significantly different (*p* ≥ 0.05).

## Data Availability

The data that support the findings of this study are available on request from the corresponding author.

## Supplementary Materials

The following supporting information can be downloaded at: https://www.mdpi.com/article/10.3390/ani16142207/s1. Text S1. Sequencing and analysis of intestinal microbiota. References [41,42,43,44] are cited in the supplementary materials. Text S2. Transcriptome sequencing analysis. Text S3. Quantitative real-time PCR analysis. References [45,46] are cited in the supplementary materials. Table S1. Primers for qPCR. Table S2. Statistic of transcriptome sequencing in *L. vannamei*.

## References

[1] Fan L., Li Q.X. (2019). Characteristics of intestinal microbiota in the pacific white shrimp *Litopenaeus vannamei* differing growth performances in the marine cultured environment. Aquaculture.

[2] Liang F., Li C., Hou T., Wen C., Kong S., Ma D., Sun C., Li S. (2020). Effects of chitosan–gentamicin conjugate supplement on non-specific immunity, aquaculture water, intestinal histology and microbiota of pacific white shrimp (*Litopenaeus vannamei*). Mar. Drugs.

[3] Liu Y., Wang W.-N., Wang A.L., Wang J.M., Sun R.Y. (2007). Effects of dietary vitamin E supplementation on antioxidant enzyme activities in *Litopenaeus vannamei* (boone, 1931) exposed to acute salinity changes. Aquaculture.

[4] Wu J., Tian S., Luo K., Zhang Y., Pan H., Zhang W., Mai K. (2022). Dietary recombinant human lysozyme improves the growth, intestinal health, immunity and disease resistance of pacific white shrimp *Litopenaeus vannamei*. Fish Shellfish Immunol..

[5] Zheng Y., Yu M., Liu Y., Su Y., Xu T., Yu M., Zhang X.H. (2016). Comparison of cultivable bacterial communities associated with pacific white shrimp (*Litopenaeus vannamei*) larvae at different health statuses and growth stages. Aquaculture.

[6] Otsuka T., Nishida S., Shibahara T., Temizoz B., Hamaguchi M., Shiroyama T., Kimura K., Miyake K., Hirata H., Mizuno Y. (2022). CpG ODN (K3)—Toll-like receptor 9 agonist—Induces Th1-type immune response and enhances cytotoxic activity in advanced lung cancer patients: A phase I study. BMC Cancer.

[7] Sun R., Wang M., Wang L., Yue F., Yi Q., Huang M., Liu R., Qiu L., Song L. (2014). The immune responses triggered by CpG ODNs in shrimp *Litopenaeus vannamei* are associated with *Lv*Tolls. Dev. Comp. Immunol..

[8] Utaynapun K., Chirapongsatonkul N., Itami T., Tantikitti C. (2016). CpG ODN mimicking CpG rich region of myxosporean *myxobolus supamattayai* stimulates innate immunity in Asian sea bass (*Lates calcarifer*) and defense against *streptococcus iniae*. Fish Shellfish Immunol..

[9] Sun R., Qiu L., Yue F., Wang L., Liu R., Zhou Z., Zhang H., Song L. (2013). Hemocytic immune responses triggered by CpG ODNs in shrimp *Litopenaeus vannamei*. Fish Shellfish Immunol..

[10] Sun R., Yue F., Qiu L., Zhang Y., Wang L., Zhou Z., Zhang H., Yi Q., Song L. (2013). The CpG ODNs enriched diets enhance the immuno-protection efficiency and growth rate of chinese mitten crab, eriocheir sinensis. Fish Shellfish Immunol..

[11] Hu F., Wang S., Hu J., Bao Z., Wang M. (2024). Comprehensive evaluation of dietary tandem CpG oligodeoxynucleotides on enhancement of antioxidant capacity, immunological parameters, and intestinal microbiota in white shrimp (*Litopenaeus vannamei*). Aquaculture.

[12] Hu F., Chen G., Hu J., Bao Z., Wang M. (2024). Transcriptome and microRNAome elucidate the mechanism underlying the immunomodulatory effects of dietary CpG oligodeoxynucleotides (CpG ODNs) in *Litopenaeus vannamei*. Aquaculture.

[13] Hu F., Wang Y., Hu J., Bao Z., Wang M. (2023). Comparative study of the impact of dietary supplementation with different types of CpG oligodeoxynucleotides (CpG ODNs) on enhancing intestinal microbiota diversity, antioxidant capacity, and immune-related gene expression profiles in pacific white shrimp (*Litopenaeus vannamei*). Front. Immunol..

[14] Zhang S., Shi L., Lu K., Li H., Wang S., He J., Li C. (2016). Cloning, identification and functional analysis of a β-catenin homologue from pacific white shrimp, *Litopenaeus vannamei*. Fish Shellfish Immunol..

[15] Jiang J., Wu X.Y., Zhou X.Q., Feng L., Liu Y., Jiang W.-D., Wu P., Zhao Y. (2016). Effects of dietary curcumin supplementation on growth performance, intestinal digestive enzyme activities and antioxidant capacity of crucian carp *Carassius auratus*. Aquaculture.

[16] Li X., Wu X., Li X., Zhu T., Zhu Y., Chen Y., Wu X., Yang D. (2023). Effects of water temperature on growth performance, digestive enzymes activities, and serum indices of juvenile *Coreius guichenoti*. J. Therm. Biol..

[17] Lou G., Guo Y., Liu X., Xiao X., Zhu X., Jiang N., Ge R., Lin Y., Lan Y., Chen X. (2025). Dietary synthetic astaxanthin and natural astaxanthin from *Haematococcus pluvialis* and *Phaffia Rhodozyma* improves the growth, antioxidant capacity, innate immunity, and pigmentation of pacific white shrimp (*Litopenaeus vannamei*). Aquacult. Nutr..

[18] Sivagnanavelmurugan M., Thaddaeus B.J., Palavesam A., Immanuel G. (2014). Dietary effect of sargassum wightii fucoidan to enhance growth, prophenoloxidase gene expression of penaeus monodon and immune resistance to *Vibrio parahaemolyticus*. Fish Shellfish Immunol..

[19] Kumar R., Huang J.Y., Ng Y.S., Chen C.Y., Wang H.C. (2022). The regulation of shrimp metabolism by the white spot syndrome virus (WSSV). Rev. Aquacult..

[20] Cox N., De Swaef E., Corteel M., Van Den Broeck W., Bossier P., Dantas-Lima J.J., Nauwynck H.J. (2023). The way of water: Unravelling white spot syndrome virus (WSSV) transmission dynamics in *Litopenaeus vannamei* shrimp. Viruses.

[21] Nguyen T.V., Alfaro A., Arroyo B.B., Leon J.A.R., Sonnenholzner S. (2021). Metabolic responses of penaeid shrimp to acute hepatopancreatic necrosis disease caused by *Vibrio parahaemolyticus*. Aquaculture.

[22] Lomelí-Álvarez M.F., Escamilla-Montes R., Diarte-Plata G., Guo X., Fierro-Coronado J.A., Rubio-Luque A.M., Vega-Carranza A.S., González A.L. (2026). Dietary and water probiotics enhance immunity, modulate microbiota, and increase survival of *Penaeus vannamei* challenged with *Vibrio parahaemolyticus*. Braz. J. Microbiol..

[23] Holt C.C., Bass D., Stentiford G.D., Van Der Giezen M. (2021). Understanding the role of the shrimp gut microbiome in health and disease. J. Invertebr. Pathol..

[24] Duan Y., Wang Y., Dong H., Ding X., Liu Q., Li H., Zhang J., Xiong D. (2018). Changes in the intestine microbial, digestive, and immune-related genes of *Litopenaeus vannamei* in response to dietary probiotic clostridium butyricum supplementation. Front. Microbiol..

[25] Kurniawinata M.I., Sukenda S., Wahjuningrum D., Widanarni W. (2022). Bacterial diversity and community composition in the gut and rearing water of pacific white shrimp *Penaeus vannamei* during an outbreak of white feces disease. Aquaculture.

[26] Wang H., Hu X., Chen J., Hu N., Yuan H., Tan B., Shi L., Zhang S. (2025). Effects of dietary cottonseed protein concentrate on growth performance, immunity, digestibility, and intestinal microbiota of *Penaeus vannamei* under different salinities. Aquaculture.

[27] Chen J., Wang H., Yuan H., Hu N., Zheng Y., Tan B., Shi L., Zhang S. (2024). Tapping chlorella vulgaris potential for enhanced growth, immunity, digestion, microbiota, and immunometabolism in *Litopenaeus vannamei* feeding across varied salinities. Aquaculture.

[28] Baker-Austin C., Oliver J.D., Alam M., Ali A., Waldor M.K., Qadri F., Martinez-Urtaza J. (2018). *Vibrio* spp. Infections. Nat. Rev. Dis. Prim..

[29] Liao G., Wu Q., Mo B., Zhou J., Li J., Zou J., Fan L. (2022). Intestinal morphology and microflora to vibrio alginolyticus in pacific white shrimp (*Litopenaeus vannamei*). Fish Shellfish Immunol..

[30] Liu F., Liu G., Li F. (2016). Characterization of two pathogenic photobacterium strains isolated from exopalaemon carinicauda causing mortality of shrimp. Aquaculture.

[31] Zhou R., Weng S., He J. (2025). Bacterial infection disrupts the intestinal bacterial community and facilitates the enrichment of pathogenic bacteria in the intestines of *Penaeus vannamei*. Microorganisms.

[32] Chen H., Zhang F., Yu J., Chen R., Zhang D., Chen C., Wang K. (2025). Divergence patterns of bacterial communities between larviculture systems of two *Penaeus vannamei* strains with distinct culture traits. Aquaculture.

[33] Xv Z., Chen S., Song G., Hu H., Lin S., Long Y. (2024). Biochemical, histological and transcriptomic analyses for the immunological organs provide insights into heat stress-induced disease susceptibility in *Largemouth bass*. Sci. Total Environ..

[34] Duan Y., Xiong D., Wang Y., Li H., Dong H., Zhang J. (2021). Toxic effects of ammonia and thermal stress on the intestinal microbiota and transcriptomic and metabolomic responses of *Litopenaeus vannamei*. Sci. Total Environ..

[35] Chen Y., Wu X., Lai J., Liu Y., Song M., Li F., Gong Q. (2023). Integrated biochemical, transcriptomic and metabolomic analyses provide insight into heat stress response in yangtze sturgeon (*Acipenser dabryanus*). Ecotoxicol. Environ. Saf..

[36] Huang W., Ren C., Li H., Huo D., Wang Y., Jiang X., Tian Y., Luo P., Chen T., Hu C. (2017). Transcriptomic analyses on muscle tissues of *Litopenaeus vannamei* provide the first profile insight into the response to low temperature stress. PLoS ONE.

[37] Yin X., Zhuang X., Liao M., Huang L., Cui Q., Liu C., Dong W., Wang F., Liu Y., Wang W. (2022). Transcriptome analysis of pacific white shrimp (*Litopenaeus vannamei*) hepatopancreas challenged by vibrio alginolyticus reveals lipid metabolic disturbance. Fish Shellfish Immunol..

[38] Sarapultsev A., Gusev E., Komelkova M., Utepova I., Luo S., Hu D. (2023). JAK-STAT signaling in inflammation and stress-related diseases: Implications for therapeutic interventions. Mol. Biomed..

[39] Joshi T., Singh A.K., Haratipour P., Sah A.N., Pandey A.K., Naseri R., Juyal V., Farzaei M.H. (2019). Targeting AMPK signaling pathway by natural products for treatment of diabetes mellitus and its complications. J. Cell. Physiol..

[40] Chen Z., Yang Q., He G.-W. (2026). LKB1–AMPK signaling pathway in cardiovascular and other diseases. MedComm.

[41] Bolger A.M., Lohse M., Usadel B. (2014). Trimmomatic: A flexible trimmer for Illumina sequence data. Bioinformatics.

[42] Caporaso J.G., Kuczynski J., Stombaugh J., Bittinger K., Bushman F.D., Costello E.K., Fierer N., Pena A.G., Goodrich J.K., Gordon J.I. (2010). QIIME allows analysis of high-throughput community sequencing data. Nat. Methods.

[43] Rognes T., Flouri T., Nichols B., Quince C., Mahe F. (2016). VSEARCH: A versatile open source tool for metagenomics. PeerJ.

[44] Wang Q., Garrity G.M., Tiedje J.M., Cole J.R. (2007). Naive Bayesian classifier for rapid assignment of rRNA sequences into the new bacterial taxonomy. Appl. Environ. Microb..

[45] Yu L., Deng J., Shi X., Liu C., Yu K., Zhou B. (2010). Exposure to DE-71 alters thyroid hormone levels and gene transcription in the hypothalamic-pituitary-thyroid axis of zebrafish larvae. Aquat. Toxicol..

[46] Livak K.J., Schmittgen T.D. (2001). Analysis of relative gene expression data using real-time quantitative PCR and the 2(-Delta Delta C(T)) Method. Methods.

