# Supplementary material for: Effects of Dietary CpG Oligodeoxynucleotides (CpG ODNs) Supplementation Levels on Growth Performance, Immunity, Digestive Capacity, Intestinal Microbiota, and Transcriptomic Response in Litopenaeus vannamei
Source: Animals (Basel). 2026 Jul 16;16(14):2207. doi: 10.3390/ani16142207 (PMC13404183; doi:10.3390/ani16142207)
Supplement: Supplementary file 1 [file animals-16-02207-s001.zip › animals-4418585-supplementary.pdf]

Supplementary material

Effects of dietary CpG oligodeoxynucleotide (CpG ODN) supplementation levels on growth performance, immunity, digestive capacity, intestinal microbiota, and transcriptomic response in *Litopenaeus vannamei*

Hongming Wang[1#], Cuihong Hou[1#], Yudong Zheng[1], Hang Yuan[1], Beiping Tan[1,2,3 *], Lili Shi[1,2,3], Shuang Zhang[1,2,3*]

1 College of Fisheries, Guangdong Ocean University, Zhanjiang, China;

2 Key Laboratory of Aquatic, Livestock and Poultry Feed Science and Technology in South China, Ministry of Agriculture, Zhanjiang, China;

3 Aquatic Animals Precision Nutrition and High Efficiency Feed Engineering Research Center of Guangdong Province, Zhanjiang, China.

# These authors contributed equally to this work and should be considered co-first authors.

* Correspondence: zshuang@gdou.edu.cn (Shuang Zhang), bptan@126.com (Beiping Tan)

Number of pages: 7; number of Texts: 3; number of Tables: 2.

Text S1. Sequencing and analysis of intestinal microbiota

Total genomic DNA of intestinal contents was extracted using DNA Extraction Kit (Magen, China) following the manufacturer's instructions. Concentration and quality of DNA was verified by using NanoDrop2100 (Thermo) and agarose gel electrophoresis. The genome DNA was used as template for PCR amplification (Takara, Japan) with the bacteria 16S rRNA V3-V4 primers following the manufacturer's instructions. The PCR products were detected by electrophoresis (Tanon, China), purified using magnetic beads (Tiangen, China) according to the manufacturer's instructions and used as second-round PCR template for second-round PCR amplification, detected again by electrophoresis and purified using magnetic beads. After quantified, the PCR products were mixed in equal amounts according to the concentration and sequenced.

Fastq format raw sequencing data were obtained. The paired-end reads were de-hybridized using Trimmomatic software (Bolger et al., 2014). Also, UCHIME was used to detect and remove the chimeric sequences from the sequences. Then, sequences were grouped into operational taxonomic units (OTUs) based on sequence similarity using Vsearch software (Rognes et al., 2016), sequences with parameters of sequence similarity greater than or equal to 97 % were grouped into one OTU unit. Representative sequences for each OTU were selected using the QIIME package (Caporaso et al., 2010) and annotated using the Silva database using the RDP classifier software (Wang et al., 2007), confidence intervals greater than 0.7 were retained.

Principal coordinate analysis (PCoA) was conducted with R language based on binary jaccard algorithm. ANOVA analysis was used to analysis the differences groups. Alpha diversity indices, including observed species, Shannon, Simpson, Chao1, and ACE, were calculated using QIIME software. Functional profiles of microbial communities were predicted using Tax4Fun based on KEGG pathway.

References:

Bolger, A.M., Lohse, M., Usadel, B., 2014. Trimmomatic: a flexible trimmer for Illumina sequence data. Bioinformatics 30(15), 2114-2120. https://doi.org/10.1093/bioinformatics/btu170.

Caporaso, J.G., Kuczynski, J., Stombaugh, J., Bittinger, K., Bushman, F.D., Costello, E.K., Fierer, N., Pena, A.G., Goodrich, J.K., Gordon, J.I., Huttley, G.A., Kelley, S.T., Knights, D., Koenig, J.E., Ley, R.E., Lozupone, C.A., McDonald, D., Muegge, B.D., Pirrung, M., Reeder, J., Sevinsky, J.R., Turnbaugh, P.J., Walters, W.A., Widmann, J., Yatsunenko, T., Zaneveld, J., Knight, R., 2010. QIIME allows analysis of high-throughput community sequencing data. Nat Methods 7(5), 335-336. https://doi.org/10.1038/nmeth.f.303.

Rognes, T., Flouri, T., Nichols, B., Quince, C., Mahe, F., 2016. VSEARCH: a versatile open source tool for metagenomics. PeerJ 4, e2584. https://doi.org/10.7717/peerj.2584.

Wang, Q., Garrity, G.M., Tiedje, J.M., Cole, J.R., 2007. Naive Bayesian classifier for rapid assignment of rRNA sequences into the new bacterial taxonomy. Appl Environ Microb 73(16), 5261-5267. https://doi.org/10.1128/Aem.00062-07.

Text S2. Transcriptome sequencing analysis

Hemocyte samples stored at −80 °C were thawed on ice, and total RNA was extracted using a tissue RNA extraction kit (TransGen Biotech, China) according to the manufacturer's instructions (n = 3). Total RNA was isolated from the samples using the TRIzol method. mRNA was purified with oligo(dT) magnetic beads and then fragmented into short fragments using fragmentation buffer. The fragmented mRNA was used as a template for first-strand cDNA synthesis with random hexamer primers. Second-strand cDNA was subsequently synthesized in the presence of dNTPs, buffer, DNA polymerase I, and RNase H. The resulting cDNA fragments were purified with Agencourt AMPure XP beads, followed by end repair, A-tailing, adapter ligation, and elution with EB buffer. Suitable target fragments were recovered by agarose gel electrophoresis, and PCR amplification was performed to complete library construction. The prepared libraries were sequenced using the Illumina HiSeq 2000 platform. Raw sequencing reads were subjected to quality control before downstream analysis. Reads containing adapter sequences, poly-A sequences, more than 10% unknown bases (N), or low-quality bases, defined as reads in which bases with $Q \leq 20$ accounted for more than 50% of the total read length, were removed. The remaining clean reads were aligned to the Pacific white shrimp reference genome (NCBI Genome database ID: 10710) using HISAT software. Transcript abundance was calculated as fragments per kilobase of transcript per million mapped reads (FPKM), as described previously. Differences in gene expression between the treatment and control groups were evaluated using the log2 fold change [log2(FC)]. The resulting statistical values were adjusted using the false discovery rate (FDR). Genes with $|\log2(FC)| > 1$ and $FDR < 0.05$ were identified as differentially expressed genes (DEGs). Functional annotation and enrichment analyses of DEGs were conducted using the Gene Ontology (GO) and Kyoto Encyclopedia of Genes and Genomes (KEGG) databases. The raw sequencing data have been deposited in the NCBI BioProject database under accession number PRJNA816062 for *L. vannamei*.

Text S3. Quantitative real-time PCR analysis

Samples (n = 3 replicates for each treatment) were collected from each treatment to extract total RNA following the protocol of Yu et al. (2010). RNA quality was assessed using 1% agarose electrophoresis and by measuring the 260/280 nm absorbance ratios. After purification using DNase I (Promega), to remove genomic DNA contamination, the total RNA was reverse-transcribed into cDNA and the gene transcription levels were analyzed using a SYBR Green PCR kit (Toyobo, Osaka, Japan) on an ABI PRISM 7300 Sequence Detector system (Perkin-Elmer, Applied Biosystems). The program was 95 °C for 10 min, followed by 40 cycles at 95 °C for 15 s, 60 °C for 20 s, and 72 °C for 45 s. The gene primer sequences were obtained from the literature or identified using the Primer5 software (Table S1). The transcriptional stability of 12 candidate genes was analyzed with qPCR and geNorm software, and *β-actin* was determined to be stable in response to all treatment groups. Target genes' transcription levels were normalized to that of *EFIα*by using the $2^{-\Delta\Delta Ct}$ method (Livak and Schmittgen, 2001).

References:

(1) Yu L, Deng J, Shi X, Liu C, Yu K, Zhou B. Exposure to DE-71 alters thyroid hormone levels and gene transcription in the hypothalamic-pituitary-thyroid axis of zebrafish larvae. Aquat Toxicol 2010; 97: 226-33.

(2) Livak KJ, Schmittgen TD. Analysis of relative gene expression data using real-time quantitative PCR and the 2(-Delta Delta C(T)) Method. Methods 2001; 25: 402-8.

Table S1 Primers for qPCR

Gene names	Gene ID No.	Primers	Sequences (5'-3')
EFIα	GU136229	F-EFIα	GTATTGGAACAGTGCCCGTG
		R-EF1α	TCACCAGGGACAGCCTCAGTA
ATP6	ncbi_113799959	F-ATP6	TTCGCAACGCAGGTGTATGT
		R-ATP6	CAGGGAAGAGAGCGGTGTCA
Rrp7a	ncbi_113802969	F-Rrp7a	GCGTTGATGACCAAACCATAAGT
		R-Rrp7a	GACCACTGCGAGTAGAGAGACCT
FUBP3	ncbi_113802684	F-FUBP3	ATCTTCGCTGGCTCTTACGC
		R-FUBP3	GGTCTGTTTGGTCTATTGGGTCT
PCK2	ncbi_113819349	F-PCK2	CGCAGGGGGTCAAGAAATA
		R-PCK2	CCCACGCACTCCACTTTGT
Ino80c	ncbi_113802527	F-Ino80c	GGTTCGCAACGCAGGTGTA
		R-Ino80c	CACGGACGGAGTGATGGAGA
eif3k	ncbi_113826211	F-eif3k	AATAAGTACGGCTGGAAGGAGGT
		R-eif3k	GCCATAATGCCAGCCACAG
hum-6	ncbi_113805391	F-hum-6	GCAACGAAATCTACGTCCAGTG
		R-hum-6	GCATTAGCCAAAGCCGTTC
chmp1a	ncbi_113814455	F-chmp1a	CAACCCTTAGCACCCCCAA
		R-chmp1a	TTTCACCAACGGCGGAGA
MRPL54	ncbi_113802973	F-27364	TGAGTTCACGCCAGACCGA
		R-27364	ATGAGAACCTCGTTGGCTGAC
ATP8	ncbi_113823947	F-ATP8	CTTTTTCACGGACGCAACC
		R-ATP8	TCAGATTCCACCCTCGTCAA
PPAF3	ncbi_113800184	F-PPAF3	CGCACTCATAAAGACCATCCA
		R-PPAF3	CTCTCGTTGTGGAGTTGACCTT
mthl11	ncbi_113800301	F-1928	ACAGCAAAAGGAACCAGAGAGTG
		R-1928	AACTTGCTGTCCCCGCTCA

Notes: Eukaryotic translation elongation factor 1 alpha, *EFIα*; ATP synthase F0 subunit 6, *MBATP6*; inter-alpha-trypsin inhibitor heavy chain H4-like, *Rrp7a*; glutathione S-transferase alpha 3-like, *FUBP3*; C-type lectin receptor B like, *PCK2*; cytochrome P450 2K4-like, *Ino80c*; tripartite motif-containing protein 14-like, *eif3k*; zinc finger protein 177-like, *hum-6*; ATP-binding cassette sub-family B member 10, mitochondrial-like, *ATP8*; S100 calcium binding protein A11-like, *mthl11.*

Table S2 Statistic of transcriptome sequencing in *L. vannamei*.

Items	C0	C0.1	C0.4	C1.6	C6.4	C25.6
Total raw reads	51303154	46593710	47323722	41223582	42941532	37082092
Total clean reads	51130138 (99.66%)	46462596 (99.72%)	47198644 (99.74%)	41117294 (99.74%)	42882960 (99.86%)	36981740 (99.73%)
GC Percentage (bp)	4105704147 (53.35%)	369126532 1 (52.81%)	379108969 2 (53.41%)	325180958 1 (52.59%)	320753697 0 (49.80%)	293105078 0 (52.69%)
Q20% (bp)	7479501555 (97.19%)	679149030 3 (97.17%)	694960904 5 (97.90%)	601227130 4 (97.23%)	632058945 0 (98.13%)	541872968 1 (97.42%)
Q30% (bp)	7118400609 (92.50%)	646118248 0 (92.45%)	667502133 6 (94.03%)	572319579 1 (92.56%)	607289369 3 (94.28%)	517427638 9 (93.02%)
Effective reads	50552122	45150346	45783040	40692828	42342446	36610928
Total mapped	45001588 (89.02%)	40023742 (88.65%)	41260468 (90.12%)	36370521 (89.38%)	39403248 (93.06%)	32746859 (89.45%)
Exon	40655972 (90.34%)	35239889 (88.05%)	35861970 (86.92%)	33036083 (90.83%)	36953668 (93.78%)	29728852 (90.78%)
Intron	2112641 (4.69%)	2449755 (6.12%)	3001222 (7.27%)	1746990 (4.80%)	1160968 (2.95%)	1539192 (4.70%)

Note: Total raw reads, Total clean reads, GC Percentage (bp), Q20% (bp), Q30% (bp), Effective reads, Total mapped, Exon, and Intron for each group are all the average values (n=3).
